# A Summary Catalogue of Microbial Drinking Water Tests for Low and Medium Resource Settings

**DOI:** 10.3390/ijerph9051609

**Published:** 2012-05-04

**Authors:** Robert Bain, Jamie Bartram, Mark Elliott, Robert Matthews, Lanakila McMahan, Rosalind Tung, Patty Chuang, Stephen Gundry

**Affiliations:** 1 Water and Health Research Centre/Merchant Venturers Building, University of Bristol, BS8 1UB, UK; Email: robert.bain@bristol.ac.uk (R.B.); rob.matthews@bristol.ac.uk (R.M.); roztung@gmail.com (R.T.); 2 The Water Institute at UNC/University of North Carolina, Chapel Hill, North Carolina, 27599, USA; Email: jbartram@unc.edu (J.B.); elliottm@email.unc.edu (M.E.); pchuang@email.unc.edu (P.C.); 3 Department of Environmental and Occupational Health, Florida International University, Miami, Florida, 33199, USA; Email: kmcmahan@gmail.com

**Keywords:** drinking-water quality, safe water, microbial water testing, faecal indicator bacteria, *Escherichia coli*, coliform test, H_2_S test

## Abstract

Microbial drinking-water quality testing plays an essential role in measures to protect public health. However, such testing remains a significant challenge where resources are limited. With a wide variety of tests available, researchers and practitioners have expressed difficulties in selecting the most appropriate test(s) for a particular budget, application and setting. To assist the selection process we identified the characteristics associated with low and medium resource settings and we specified the basic information that is needed for different forms of water quality monitoring. We then searched for available faecal indicator bacteria tests and collated this information. In total 44 tests have been identified, 18 of which yield a presence/absence result and 26 of which provide enumeration of bacterial concentration. The suitability of each test is assessed for use in the three settings. The cost per test was found to vary from $0.60 to $5.00 for a presence/absence test and from $0.50 to $7.50 for a quantitative format, though it is likely to be only a small component of the overall costs of testing. This article presents the first comprehensive catalogue of the characteristics of available and emerging low-cost tests for faecal indicator bacteria. It will be of value to organizations responsible for monitoring national water quality, water service providers, researchers and policy makers in selecting water quality tests appropriate for a given setting and application.

## 1. Introduction

Testing water quality is a key element of drinking water safety that has been gaining increasing attention, especially in reference to the close of the Millennium Development Goals (MDG) in 2015 [1,2]. A World Health Organization (WHO) and UNICEF Task Force stated recently that it is “essential that new targets for post-2015 efforts should include water quality” [3]. Water testing plays an important role in ensuring the correct operation of water supplies, verifying the safety of drinking-water, investigating disease outbreaks, and validating processes and preventative measures. There are significant challenges in implementing comprehensive and appropriate water quality testing, particularly in low-resource settings. As a consequence, the extent and quality of the information provided by water testing is often inadequate to support effective decision-making.

Microbial contamination is responsible for the great majority of water-related health burden [4]. WHO recommends that the microbial quality of drinking-water be measured using faecal indicator bacteria, preferably *Escherichia coli*; these bacteria are chosen to indicate the presence of faecal contamination rather than identifying pathogens directly [4]. Conventionally, analyses take place in a laboratory environment using standard procedures, such as those described in the *Standard Methods for the Examination of Water and Wastewater* [5], approved by the U.S. Environmental Protection Agency or set by the International Organization for Standardization. We have restricted our analysis to tests based on culturing faecal indicator bacteria as these are likely to remain the most common methods for microbial water quality monitoring in the short- to medium-term.

Conventional laboratory methods, such as membrane filtration and multiple tube fermentation, are complex and time-consuming. They require a wide range of basic laboratory equipment and skilled personnel to achieve consistent results. Sample transportation, especially within the recommended timeframe (<24 h, preferably <6 h [6]) and temperature range (<8 °C but not frozen [5]), is often impractical. This is particularly the case for rural and dispersed populations, for which the nearest laboratory can be at a significant distance from water supplies. Where laboratories are accessible, these may be overstretched and only able to conduct infrequent testing of a limited number of supplies. As a consequence, testing in the locations with no access to resources such as reliable mains electricity or technically trained staff may be preferable. 

Several tests, most notably portable membrane filtration and the hydrogen sulphide test [7], have sought to address the challenges of using traditional methods in remote and low-resource settings. Developments in chromogenic and fluorogenic enzyme-substrate tests have also greatly expanded the range and variety of available tests. A detailed understanding of the resource requirements and information provided by these is needed in order to select an appropriate test. However, the available information on microbial water tests is poorly consolidated and in many cases difficult to access—this is particularly the case for costs and test performance. Although previous reviews [8,9,10,11,12] include a number of factors that are essential in the selection of tests for drinking-water, they are limited in scope, do not evaluate tests on a consistent basis or do not provide a side by side comparison. As a result, it can be difficult for practitioners to select tests for a particular setting, application and budget.

Our objectives were:

(1) To define, in reference to resource settings and the purposes of testing, important characteristics which should be considered when selecting a test for faecal indicator bacteria in drinking-water.
(2) To collate information on these characteristics for available water tests and assess their suitability based on the resources available in a given setting.

We have not carried out any microbiological assessments of the performance of the various tests. We expect users of our catalogue should satisfy themselves that the performance of the tests will meet their needs. Most manufacturers’ websites make available the findings of appropriate, objective studies. Consequently, we are able to assess suitability for resource settings, but not the fitness for purpose. Notwithstanding these limitations, we believe that the catalogue will be useful for an audience ranging from organizations responsible for monitoring national water quality and water service providers to researchers and policy makers.

## 2. Methods

### 2.1. Identifying Characteristics for Inclusion in the Assessment

In order to assess the applicability of individual tests, three resource settings and the main applications of water testing have been defined (Table 1 and Table 2). Important characteristics to be considered when selecting a test were then identified based on these definitions and the authors’ experience. The definitions of the resource settings focus on the available infrastructure, rather than financial and human resources.

**Table 1:** Resource settings.

Low resource	Medium resource	High resource
No laboratory. Clean space without electricity or by the water source.	Basic laboratory or clean space with electricity within 24 hours.	Modern laboratory within 24 hours, including vacuum, distilled water, fume hood and a cold supply chain. Reliable electricity.

The information provided by a monitoring program (Table 2) is influenced by two main factors, the indicator bacteria and the extent of quantification, both of which are impacted by regulatory standards. The main forms of monitoring water quality can be referred to as compliance/surveillance, depending on the agency using the data, and operational monitoring. For compliance or surveillance monitoring, regulatory approval of the test is usually required. Operational monitoring is done in the context of Water Safety Plans, a risk-based framework for managing water supplies [13]. Other uses, such as treatment efficacy testing, educational and awareness-raising, while important, typically demand lesser amounts of testing and the information requirements will need to be determined on a case by case basis. 

**Table 2:** Types of monitoring and information needs.

Type	Definition	Information needed		
		Indicator ^1^	Quantification	Regulatory Approval
Compliance or Surveillance	Compliance monitoring is conducted by water service providers to demonstrate that water meets the regulatory standards	As regulated	As regulated	Required
	Surveillance monitoring is conducted by an independent agency to ensure water is safe	Health based, Usually TC and/or EC	Desirable, ideally with range depending on health risk	
Operational	The monitoring of operational parameters to ensure treatment is functioning	Operational parameter, often TC	Desirable	Desirable
Other	Examples include research into water treatment efficacy testing, educational and awareness-raising or controlling for water quality as part of a study	Varies	Varies, though often desirable	Desirable

^1 ^EC—*Escherichia coli*, TC—Total coliforms.

### 2.2. Finding and Assessing Tests

A preliminary set of microbial water tests was identified by the authors and colleagues based on experience of using and developing microbial drinking-water tests. This was supplemented by a review of the literature and internet searches. For the internet searches, keywords included the names of the indicator bacteria groups, chemical substrates used to detect these (such as β-glucuronides and β-galactosides) [14,15], generic terms such as ‘water test’, the names of previously identified tests and their combinations. We refer to ‘test’ as the consumables used per analysis, a ‘kit’ refers to the test and required equipment. The list of water quality tests was reviewed by colleagues in industry, academia and practice to ensure that it was comprehensive.

Microbial water tests have been included if:

(1) The tests are in common use or widely known to be in the latter stages of development.
(2) The tests are relatively inexpensive (<$10 per test and <$5,000 for specialized equipment).
(3) They detect faecal indicator bacteria typically used for drinking-water analysis, namely *Escherichia coli (E. coli)*, total coliforms, thermotolerant coliforms, or hydrogen sulphide (H_2_S) producing bacteria.
(4) The volume of the sample is least 1 mL, providing a lower detection limit of ≤100 indicator organisms per 100 mL.

For each water test identified in this review, details were recorded for the characteristics defined in Table 3. These were obtained from a variety of sources, including test protocols and direct communication with manufacturers. The suitability of each test for a given resource setting was assessed based on the resources required to conduct each test according to its standard protocol and in reference to Table 1. Tests that only require resources which are likely to be available in a given setting are “recommended” (green). Where a test cannot be recommended, but modifications to the procedure could be made to overcome the resource requirements, the test is considered “not ideal” (brown). Tests that would be very challenging to conduct in a setting or may be unsafe have been labeled “not suitable” (black).

A draft version of the assessment was presented at the *Water and Health: Where Science Meets Policy* conference at the University of North Carolina [16]. Questions were raised regarding the dependence of sanitary significance on source type and what transport restrictions applied to the various tests. As a result of feedback, the shelf life columns in Table 4 and Table 5 were revised to include information on specific storage temperature ranges; these have been included in “other” characteristics. The groupings used at the conference were expanded upon in order to provide better differentiation between similar tests. These revised versions of the tables were subsequently provided to manufacturers for comments and corrections. Manufacturers were also asked for suggestions on how these tests might be applied in low and medium resource settings, for example by using alternative equipment or procedures. All 20 manufacturers of tests in the list were contacted; 14 responded with further information on their tests or comments and corrections. 

## 3. Results

### 3.1. Characteristics Included in the Assessment

The characteristics included in the assessment are listed in Table 3

**Table 3:** Definition of characteristics.

Characteristic	Definition
Cost per test ^1,2^	These costs are based on the purchase of 400 to 500 tests. They do not include delivery or importation costs.
Cost of specialized equipment ^1^	Equipment which is needed for this particular test which would not typically be available in a laboratory. The cost is based on a single unit of each piece of durable equipment or in the case of glassware, the quantity typically used for a single analysis.
Analysis time ^3^	Time taken to conduct a single test, excluding the time required for transport and incubation. This includes preparation of media, interpretation of results and appropriate disposal.
Trained technician	A trained technician is required if training is at least one day, for example if standard microbiological techniques are needed.
Controlled incubation	Required if specified in the standard procedure for the test.
Ultraviolet light	Required for the detection of fluorogenic substrates.
Sterilization/disinfection	Required unless the test contains an integral disinfectant.
Deionised water	Required for some tests, especially membrane filtration where water samples may require dilution.
Cold storage	Required if the test needs to be stored below room temperature.
Transport	Required if tests cannot be conducted at the water source or if tests require a vehicle
Disposal ^2^	Amount of waste generated by each test, including sample collection vessels.
Sample volume meeting WHO Guidelines	The test is able to satisfy the sample volume aspect of the WHO guidelines “none detected in 100 mL”.
Undiluted range ^4^	The lower and upper detection limit for the concentration of bacteria when no dilution is performed and the maximum sample volume is analysed.
Precision ^5^	Relative assessment of the precision of quantitative estimates over the range.
Indicator	The indicator bacteria used to identify fecal contamination of drinking-water.
Sanitary significance ^6^	Relative assessment of the relationship of the indicator to *E. coli*.
Standard or approved 7	Whether the test has been approved by the U.S. EPA, is included in the Standard Methods for the Examination of Water and Wasterwater or is an International Organization for Standardisation standard.
Time to result	The minimum incubation time stated to obtain the final results from a test. A range is given for devices where incubation time varies, for example depending on the concentration of bacteria in the sample or the incubation temperature.
Shelf life	Shelf life from manufacture, based on dehydrated media where available.
Storage temperature	Recommended long-term storage temperature of test or medium.

^1 ^Costs were obtained from websites, catalogues or quotations from manufacturer or suppliers. For non-proprietary tests costs were estimated based on lowest cost consumables from Sigma Aldrich or Beckton Dickinson. Where a separate sample vessel or disposable pipette is required, these have been added at a cost of $0.50 and $0.10 respectively. Current exchange rates were obtained from xe.com (accessed 28th Feb 2012) to derive costs in the same currency, USD. The cost per test and cost of specialized equipment have been rounded to the nearest $0.10 and $100 respectively; ^2 ^The volume of waste and the cost per test for laboratory methods are based on reusable components; ^3 ^Analysis time assumes that only a small number of tests are conducted on a single day (<10); ^4 ^A single presence/absence test does not provide a quantification of the contamination level and as such a range has not been defined for these; instead we provide the lower detection limit based on the total volume tested. By dividing a sample into subsamples, most probable number devices can yield a statistical estimate of the level of contamination, called the Most Probable Number or MPN [17]. A MATLAB program (version R2010b) was used to evaluate the highest MPN where a manufacturer’s MPN table was not available or appeared to be inconsistent with the specified volumes; this follows the method recommended by the U.S. FDA [18]; ^5 ^For MPN tests the precision has been assessed based on the calculated or published MPN tables and colony count tests have been assigned as “best”; ^6 ^In order of decreasing specificity to *E. coli*, we have assigned the following as: Thermotolerant coliforms, “good”; Total coliforms and H_2_S production, “moderate”; ^7 ^These approvals or standards are the basis of regulations in many, but not all, countries.

### 3.2. Summary Assessment

Table 4 provides a summary of the main categories of tests and how these compare for the range of characteristics we have assessed. Table 5 lists the manufacturers of these tests. In total, 44 tests were identified in this study. Presence/absence (PA) tests are covered in the first section of Table 4. Two main approaches are used to enumerate fecal indicator bacteria: colony counts and the most probable number (MPN); these have been used to group quantitative tests. Colony counts are achieved by plating, filtration or immobilization of the indicator bacteria within a gel. MPN tests rely on sample division or dilution and a statistical method to estimate the level of contamination.

All PA tests can produce a quantitative result if a number of replicates are used or equivalently the sample is subdivided, with or without dilution; this is the principle behind MPN tests. However, the precision and range will be limited unless several replicates at different dilutions or volumes are used. Conversely, all quantitative tests can be interpreted in a PA manner, with the total volume of original sample determining the limit of detection.

**Table 4:** Catalogue of microbial drinking water tests.

Type		Product	Resources required											Information provided						Other			Settings		
			Cost per test ^1^	Cost of specialist equipment ^2^	Analysis time (min)	Trained technician	Controlled incubation	Ultraviolet light	Sterlisation/disinfection	Deionised water	Cold storage	Transport	Disposal	Sample volume meeting WHO Guideline (100mL)	Undiluted range (Per 100 mL)	Precision	Indicator	Sanitary significance	Standard or approved	Time to result (hrs)	Shelf-life (mths)	Temperature (°C)	Low resource	Medium resource	High resource
**Presence Absence**	**Hydrogen sulphide**	PathoScreen^TM^	$0.60	$0	<5				x				S	-	>5	N/A	H_2_S	+		24–72	12	RT			
		LTEK H_2_S 20 mL	$0.80	$0	<5				x				S	-	>5	N/A	H_2_S	+		24–72	24	RT			
		HiWater^TM^	$2.40	$100	<5				x				M	+	>1	N/A	H_2_S	+		24–72	24	RT			
		LTEK H_2_S 100 mL	$1.50	$0	<5				x				M	-	>5	N/A	H_2_S	+		24–72	12	RT			
		Local manufacture	∆	$0	<5				x				S	∆	∆	N/A	H_2_S	+		24–72	∆	RT			
	**Total**	Lamotte® Coliform	$1.20	$0	<5				x				S	-	>10	N/A	TC	+		44–48	24	RT			
	**Coliform**	Rapid HiColiform^TM^	$0.80	$100	<5		x		x			x	M	+	>1	N/A	TC	+		24	36	2-8			
	***E. coli*** ** and Total coliform**	Colilert® 10 mL	$1.50	$100	<5		x	x	x			x	S	-	>10	N/A	TC&EC	+++	x	24	12	4–30			
		Colilert® 100 mL	$5.00	$100	<5		x	x	x			x	M	+	>1	N/A	TC&EC	+++	x	24	12	4–30			
		Colisure®	$5.00	$100	<5		x	x	x			x	M	+	>1	N/A	TC&EC	+++	x	24	12	2–25			
		Colilert® 18	$5.00	$100	<5		x	x	x			x	M	+	>1	N/A	TC&EC	+++	x	18	15	2–25			
		Modified Colitag^TM^	$4.50	$100	<5		x	x	x			x	M	+	>1	N/A	TC&EC	+++	x	16	22	4–30			
		Watercheck^TM^ [BWB] ^3^	$5.00	$2,700	<5		x	x	x			x	M	+	>1	N/A	TC&EC	+++		24	36	2–30			
		Readycult®	$3.00	$100	<5		x	x	x			x	M	+	>1	N/A	TC&EC	+++	x	24	36	15–25			
		E*Colite	$3.00	$100	<5		x	x				x	M	+	>1	N/A	TC&EC	+++	x	28	12	RT			
		EC Blue 100P	$3.70	$100	<5		x	x	x			x	M	+	>1	N/A	TC&EC	+++		24	12	RT			
		AquaCHROM^TM^	$2.60	$0	<5		x		x			x	M	+	>1	N/A	TC&EC	+++		18	24	15–30			
		HiSelective^TM^ *E. coli*	$2.20	$0	<5		x		x			x	M	+	>1	N/A	TC&EC	+++		24–48	12	2–8			
**Most Probable Number**	**Most Probable Number**	Compartmentalised bag test	$1.00	$0	<5								S	+	1–43	+	EC	+++		24–72	6–9	RT			
			$1.00	$0	<5								S	+	1–43	+	H_2_S	+		24–72	6–9	RT			
		Aquatest^TM^	$4.00	$100	5		x	x					M	+	1–230	+	EC	+++		24	24	RT			
		Coliplate^TM^	$7.50	$200	10	x	x	x	x			x	L	-	5–2400	+++	TC&EC	+++		24	36	2–30			
		EC BlueQuant	$5.80	$100	5	x	x	x	x			x	L	+	1–1610	++	TC&EC	+++		24	12	RT			
		Multiple tube (LTB/EC-MUG)	$3.50	$200	30	x	x	x	x	x		x	S	∆	∆	∆	EC	+++	x	48	36	RT			
		Multiple tube (LTB/BGLB)	$2.10	$200	30	x	x		x	x		x	S	∆	∆	∆	TC	+	x	36	36	RT			
		Colitag/iMPN1600	$5.77	$0	10	x	x	x	x			x	L	+	1–1600	++	TC&EC	+++	?	16	22	4–30			
		Colilert/Quanti-Tray®	$5.50	$4,100	10	x	x	x	x			x	L	+	1–200	+++	TC&EC	+++	x	18/24	12	2–25			
		Colilert/Quanti-Tray® 2000	$6.00	$4,100	10	x	x	x	x			x	L	+	1–2419	+++	TC&EC	+++	x	18/24	12	2–25			
**Colony Count**	**Plate Methods**	Petrifilm^TM ^E.coli/coliform	$1.30	$100	<5		x		x		x	x	S	-	100–5000	+++	TC&EC	+++		24	18	≤8			
		Petrifilm^TM ^Aqua Coliform	$0.70	$100	<5		x		x		x	x	S	-	100–5000	+++	TC	+		24	18	≤8			
		CHROMagar^TM^ ECC	$0.80	$100	15	x	x		x			x	S	-	100–5000	+++	TC&EC	+++		24	36	15–30			
		Compact Dry EC^TM^	$1.50	$0	<5		x		x			x	S	-	100–5000	+++	TC&EC	+++		24	24	1–30			
	**Gel based**	Coliscan Easygel	$2.20	$0	5	x	x		x		x	x	M	-	20–1000	+++	TC&EC	+++	x	24	12	<0			
		ColiGel/PathoGel ^6^	$3.50	$100	5		x	x				x	M	+	1–100 (TC) 1–25 (EC)	+++	TC&EC	+++		28	12	RT			
**Colony Count**	**Membrane Filtration ^4^**	Portable kit/LSB ^5^	$0.50	$2,700	20	x	x		x	x		x	S	∆	∆	+++	TC/TTC	++		24	48	RT			
		Portable kit/m-coliblue 24^TM^	$2.50	$4,000	15	x	x		x	x	x	x	M	∆	∆	+++	TC/TTC	+++	x	24	12	2–8			
		m-Coliblue 24^TM^	$2.50	$2,500	15	x	x		x	x	x	x	M	∆	∆	+++	TC&EC	+++	x	24	12	2–8			
		Coliscan MF^TM^	$2.20	$2,500	15	x	x		x	x	x	x	M	∆	∆	+++	TC&EC	+++		24	12	<0			
		m-Endo	$1.50	$2,500	15	x	x		x	x		x	M	∆	∆	+++	TC	+	x	24	48	RT			
		m-FC	$1.50	$2,500	15	x	x		x	x		x	M	∆	∆	+++	TTC	++	x	24	48	RT			
		CHROMagar^TM^ Liquid ECC	$1.10	$2,500	15	x	x		x	x		x	M	∆	∆	+++	TC&EC	+++		24	36	15-30			
		CHROMagar^TM^ ECC	$1.30	$2,500	15	x	x		x	x		x	M	∆	∆	+++	TC&EC	+++		24	36	15-30			
		MI Agar	$1.70	$2,500	15	x	x		x	x		x	M	∆	∆	+++	TC&EC	+++	x	24	36	RT			
		Chromocult	$1.20	$2,500	15	x	x		x	x		x	M	∆	∆	+++	TC&EC	+++	x	24	60	RT			
		Rapid *E.coli*	?	$2,500	15	x	x		x	x		x	M	∆	∆	+++	TC&EC	+++		24	?	?			

^1 ^Costs are known to vary greatly from one location to another, depending on supplier, importation taxes and delivery charges. Where not included in the kit, sample collection vessels are required and add an additional $0.50 per test. For plate methods a disposable pipette at $0.10 has been added; ^2^ Specific equipment costs are based on: UV torch ($100), membrane filtration assembly, including vacuum pump ($2500), glassware and racks for multiple tube fermentation ($200), IDEXX Quanti-Tray Sealer ($4000) and portable membrane filtration kits ($2700); ^3^ [BWB] refers to the Bluewater Biosciences Watercheck^TM^ and is not to be confused with the B2P version, denoted [B2P]; ^4^ Costs for membrane filtration are based on one filter. More filters may be used if water is very turbid or may be highly contaminated; ^5 ^Portable kits are available from a number of manufacturers including Wagtech, DelAgua and ELE. The cost varies depending on the kit and ranges from approximately $2500 to $5000; ^6^ PathoGel includes an indicator for H_2_S production (P/A).

**Table:** Key for Table 4

Symbol	Meaning
?	Value not known
N/A	Not applicable
x	Equipment or resource required
S	Small
M	Medium
L	Large
∆	Varies
-	No/Poor
+	Yes/Moderate
++	Good
+++	Best
TC	Total Coliforms
H_2_S	Hydrogen sulphide production
EC	*Escherichia coli*
TTC	Thermotolerant coliforms
RT	Room temperature
	Suitable for resource setting
	Not ideal for resource setting
	Not suitable for resource setting

**Table 5:** Suppliers of proprietary water tests.

Type	Product	Manufacturer	Website
Colony Count	CHROMagar™ ECC	CHROMagar	www.chromagar.com
	CHROMagar™ Liquid ECC	CHROMagar	www.chromagar.com
	Chromocult	EMD Chemicals	www.emdchemicals.com
	Coliscan Easygel	Micrology labs	www.micrologylabs.com
	Coliscan MF™	Micrology labs	www.micrologylabs.com
	Compact Dry EC™	Nissui Pharma	www.nissui-pharm.co.jp
	Portable Membrane Filtration	Delagua	www.delagua.org
	Portable Membrane Filtration	ELE	www.ele.com
	Portable Membrane Filtration	Wagtech	www.wagtech.co.uk
	Portable Membrane Filtration	Merck Millipore	www.millipore.com
	m-Coliblue 24™	Merck Millipore	www.millipore.com
	Petrifilm™ *E.coli*/Coliform Count	3M	www.3m.com
	Petrifilm™ Aqua Coliforms	3M	www.3m.com
	RAPID’*E. coli*	Bio Rad Labs	www.bio-rad.com
	ColiGel/PathoGel	Charm Sciences	www.charm.com
Most Probable Number	Aquatest™^1^	Aquatest consortium	www.bris.ac.uk/aquatest
	Colilert 10 mL	IDEXX	www.idexx.com
	Coliplate™	Bluewaterbiosciences	www.bluewaterbiosciences.com
	Compartmentalised bag test ^1^	University of North Carolina	www.unc.edu/sobseylab
	Compartmentalised bag test ^1^	University of North Carolina	www.unc.edu/sobseylab
	EC BlueQuant	Nissui Pharma	www.nissui-pharm.co.jp
	LaMotte Coliform test MPN	LaMotte	www.lamotte.com
	Modified Colitag™/iMPN1600 ^1^	CPI	www.cpiinternational.com
	Colilert/Quanti-Tray^®^ 200	IDEXX	www.idexx.com
	Colilert/Quanti-Tray^®^ 2000	IDEXX	www.idexx.com
Presence/Absence	AquaCHROM™	CHROMagar	www.chromagar.com
	Colilert^® ^10 or 100 mL	IDEXX	www.idexx.com
	Colilert^®^ 18™	IDEXX	www.idexx.com
	Colisure^®^	IDEXX	www.idexx.com
	Modified Colitag™	CPI	www.cpiinternational.com
	E*Colite	Charm Sciences	www.charm.com
	EC Blue 100P	Nissui Pharma	www.nissui-pharm.co.jp
	H_2_S test 20 or 100 mL	LTEK	www.lteksystems.com
	HiSelective™ *E. coli*	HiMedia	www.himedialabs.com
	HiWater™	HiMedia	www.himedialabs.com
	LaMotte^®^ Coliform	LaMotte	www.lamotte.com
	PathoScreen™	Hach	www.hach.com
	Rapid HiColiform™	HiMedia	www.himedialabs.com
	Readycult^®^	EMD Chemicals	www.emdchemicals.com
	Watercheck™	Bluewaterbiosciences	www.bluewaterbiosciences.com

Standard media (LTB, BGLB, EC MUG, MI Agar, m-Endo, modified m-TEC, m-FC etc.) are available from a variety of suppliers, including BD (www.bd.com), Sigma (http://www.sigmaaldrich.com); ^1^ Product not commercially available at time of publication.

## 4. Discussion

There is a wide variety of characteristics within our catalogue. We recommend that users should select a short-list of tests for further consideration, based on two criteria: (i) matching tests to resources and (ii) matching tests to applications. After selecting a shortlist for further consideration, users should consult manufacturers’ websites to review the microbiological performance assessments that have been carried out to ensure that the chosen products will provide appropriate sensitivity and specificity for the target application.

### 4.1. Matching Tests to Resource Settings

When considering the resource constraints, is it valuable to consider a number of alternative testing arrangements which could include: transport of the sample to a fixed laboratory, mobile field testing laboratory, decentralized onsite testing, and sample preparation onsite followed by incubation in a laboratory. If the testing forms part of a longer term monitoring system, sampling strategies including screening and/or combining complementary tests should be considered. Decisions on where and how to conduct the testing may be equally, or more important, than the cost per test [19]. This will be the case especially when the costs of transport, labor, and setting up, equipping and maintaining laboratories are taken into account. As such, whether testing will be taking place at the source using a portable kit, in a nearby health clinic or district laboratory warrants careful consideration. 

The use of many of the tests included in this assessment in low- and middle-resource settings is limited by equipment required to conduct the tests. This is particularly the case if a decentralized approach to testing is adopted, wherein many full sets of equipment are needed. By selecting lower-cost alternatives to standard equipment, or modifying testing methods (Table 6), significant savings on the cost of equipment may be possible. In most cases, the extent to which performance is compromised by these adaptations is not well understood. Despite these options, cold storage, safe handling and disposal, training, and temperature control during incubation or, where required, sample transport remain barriers to testing in low-resource settings. Furthermore, transport restrictions are known to apply to the consumables required for some tests, such as methanol for portable membrane filtration. 

**Table 6:** Alternatives to standard equipment and methods.

Standard ^1^	Alternative ^1^	Advantages	Limitations
**Laboratory Incubator ($$$)**	Ambient incubation (-)	Possibly acceptable in tropical climates [20] or potentially indoors	Recoveries for injured bacteria may be poor; increased and poorly defined incubation time; not applicable everywhere
	Low-cost electric incubators ($–$$) e.g., egg incubator	Good temperature control	Reliant on electricity, may not be available for higher temperatures (44.5°C for TTC)
	Body incubation (-)	Readily available	Acceptability, health and safety issues and limited number of tests
	Phase change incubator ($)	Good temperature control, only requires hot water	Requires heat source, can be bulky, particularly for many tests
**De-ioniser ($$$)**	Boiled water (-)	Readily available	May not inactivate all organisms; can concentrate chemical contaminants need to run blanks
	Steam distiller ($$)	Produces very pure water	Requires high-voltage and power; requires running water; fragile
	Autoclave (see below)	May be available or has dual use (see below)	Turbid waters may not provide suitable dilution water, especially for membrane filtration
**Membrane Filtration assembly & vacuum ($$$)**	Portable MF assembly, including hand pump or MIT D-lab kit ($–$$)	Manual, portable	Time consuming procedure Separate incubator required
**Autoclave ($$)**	Portable autoclave ($)	Portable	Requires heat source
	Pressure cooker ($)	Independent of electricity	Requires heat source
	Bleach or disinfectant ($)	Readily available, good for disinfecting waste	Handling of cultures; care must be taken in reusing components to prevent false negatives due to residual disinfectant
**Refrigerator or freezer ($$)**	Storage at room temperature (-)	Independence from electricity	Shelf-life unclear for many media, particularly hydrated media; samples cannot be retained for subsequent analysis
**Sample transport on ice**	Ambient temperature transport	Simple	Potential population increase or die-off of bacteria
	Insulated box (with cool water if available)	May be better than ambient	Change in bacterial population unknown, likely to be better than ambient

^1 ^Approximate costs are: Free (-), $ (1–100), $$ (100–1,000), $$$(1,000–10,000).

### 4.2. Matching Tests to Applications

While it is relatively straight-forward to classify tests based on suitability for resource settings and most manufacturers will provide performance statistics for sensitivity and specificity, a similar, simple classification is not possible for the suitability of tests for particular applications. The purpose of testing may need to be established on a case by case basis. In general, there are three main factors in low and medium resource settings: indicator bacteria, quantitative performance and regulatory approvals.

The choice of indicator bacteria will be influenced by the application; a distinction can be drawn between cases where presence of the indicator is evidence of faecal pollution, and therefore potential health risk, or an assessment of the efficacy of a treatment process [21]. The former requires that the indicator be ubiquitous in faeces but must not occur naturally. As some total coliforms occur naturally in the environment *E. coli*, or alternatively thermotolerant coliforms, are recommended by the WHO [4]. This is reflected by the sanitary significance column of Table 4 and Table 5. *E. coli* are also used for treatment assessment purposes, but in this context total coliforms are generally recommended [22]. Both indicators suffer from being more sensitive to disinfection processes than some pathogens [4]. Tests that detect the presence of H_2_S-producing bacteria are frequently used, particularly where resources are limited; however there is ongoing debate about their sanitary significance [23,24].

Quantitative tests are generally more expensive and require more resources. If the purpose of testing is to ascertain whether water meets national regulations (or the WHO Guidelines [4]), PA tests may be entirely adequate as long as the volume is sufficient and the test has the necessary validation and approvals. PA tests are also valuable when monitoring water supplies that are usually free of contamination. The resulting string of “non-detects” or infrequent positives gives more confidence than a single quantitative test [25]. However, if there is a need for relative prioritization (e.g., source selection) or if monitoring changes over time and there is a reasonable risk of contamination, a quantitative test will generally add value. Furthermore, the cost per analysis increases if a wide range of contamination levels are to be measured with high precision. As such, the range of a test, its lower and upper detection limits, needs careful consideration. For operational monitoring this decision should be based on an understanding of the likely levels of contamination in the sources being assessed. Guidance on the volumes which should be assessed using membrane filtration and multiple tube fermentation are available elsewhere [6,11]. It should be noted that the ranges are likely to be strongly influenced by both indicator bacteria and source type. For surveillance monitoring the testing of volumes lower than the WHO Guideline of 100 mL should only be considered if the majority of supplies are known to be contaminated. The range and precision should also be chosen with thought given to data analysis, decision-making, responsibilities and integration with existing data. 

Regulatory approvals are required for compliance and, usually, surveillance monitoring. Furthermore they provide additional reassurance of tests’ performance. We have not conducted a review of international regulations; instead, we compiled information on whether tests have obtained U.S. EPA approval or are featured in the Standard Methods [5] or standards published by the International Standards Organization. These approvals are the basis of the standards in a number of countries. 

### 4.3. Limitations

There are number of limitation to this assessment. Firstly, the full cost of testing will include a number of factors which we have not been able to take into account in this catalogue. This includes variability in the per test and equipment costs resulting from shipping and distribution. In most cases, a significant element of the overall cost of testing will be related to the resources such as labor, transport and infrastructure [19]. We have listed many of the resource requirements, but we do not calculate their associated costs; clearly this will vary considerably depending on the circumstances. Secondly, a number of characteristics were not included in this catalogue. Test performance in terms of false positive and false negative rates (or specificity and sensitivity) was not included as this information is not available for all tests and, unless a comparative study (e.g., [26]) is undertaken, these cannot be compared on a consistent basis. A review of the validations and national regulatory approvals each test has obtained was beyond the scope of this assessment. The precision of tests varies depending on the concentration of indicator bacteria. The availability of tests and equipment will vary both within and between countries; this would need to be established for a particular setting and factored into test selection. Thirdly, we do not include all microbiological growth media or tests based on the detection time for which sufficient information was not made available. Finally, we have not assessed the suitability of individual or combinations of tests for particular applications. This is because the information required depends on a number of variables (such as source type and regulatory standards) and is context specific. Moreover, ongoing debate about the sanitary significance and applicability of indicator bacteria, particularly H_2_S [23,24], limits the guidance that can be provided. 

## 5. Conclusions

Important characteristics to consider when choosing a microbial drinking-water test have been identified and this information has been compiled for 44 tests. The tabulated information should assist users in short listing tests for their particular requirements and setting. The identified tests include both presence/absence and quantitative tests for *E. coli*, total and thermotolerant coliforms, and H_2_S. The results are provided in tabular form to facilitate comparisons between tests. 

The cost per test was found to range from $0.60 to $5.00 for a presence/absence device and from $0.50 to $7.50 for a quantitative test. Although the costs of tests themselves are important, in fact they are likely to be a small component of the overall costs of testing, when the infrastructure and human resources are considered. The ability of tests to support alternative testing arrangements that reduce reliance on these resources and consequently the overall cost of testing is a key issue.

Few of the identified tests are ideal for low-resource settings if implemented according to their standard protocols. This is especially the case for quantitative tests. A number of alternative procedures which would greatly simplify testing in low-resource environments have been identified. We encourage further work to evaluate these and establish guidelines for their application. 
